# Highly specific ID-UHPLC-MS/MS method for analyzing polar and non-polar steroid hormones

**DOI:** 10.1007/s00216-026-06504-3

**Published:** 2026-04-29

**Authors:** Lumi Duke, Paul H. Kim, Alicia N. Lyle, Julianne C. Botelho, Natalie D. Shaw, Hubert W. Vesper

**Affiliations:** 1 Centers for Disease Control and Prevention, National Center for Environmental Health, 4770 Buford HWY NE, MS-F25, Atlanta, GA 30341 USA; 2 Battelle Memorial Institute, Atlanta, GA 30345 USA; 3 National Institutes of Health, National Institute of Environmental Health Sciences, Research Triangle Park, NC 27709 USA

**Keywords:** Steroid hormones, LC-MS/MS, Estrogens, Androgens, Progestogens, Liquid-liquid extraction

## Abstract

The accurate and precise measurement of endogenous steroid hormone levels in serum is essential for their use as biomarkers of endocrine and metabolic diseases in patient care and translational science. Herein, we describe a newly developed, highly accurate, and precise isotope-dilution liquid chromatography-mass spectrometry (LC-MS/MS) method capable of simultaneously measuring eight clinically relevant polar and non-polar steroid hormones in 200 µL of serum. This steroid hormone panel method uses sequential liquid-liquid extractions for the isolation of total testosterone (TT), estradiol (E2), progesterone (P4), 17-hydroxyprogesterone (17-OHP), androstenedione (AD), estrone (E1), estrone sulfate (E1S), and dehydroepiandrosterone sulfate (DHEAS) without derivatization or hydrolysis. The method demonstrated a broad analytical measurement range for all eight hormones as a result of improved selectivity and sensitivity, making it suitable for the analysis of general population serum samples, including postmenopausal women and children. The total imprecision, expressed as coefficients of variation, was evaluated at three levels for each analyte and ranged from 3.5 to 10.7%. The mean bias to well-established secondary reference materials was −1.00% for TT and 1.91% for E2. When applied to 268 commercially sourced serum samples from men, women, and children, only 2% of measurement results were below the limits of detection; therefore, this method was deemed suitable for measurement of eight steroid hormones across the general population. The establishment of this new steroid hormone panel LC-MS/MS method will facilitate investigations of associations between health disorders and individualized steroid profiles, which can be utilized for evaluations at the individual or population levels.

**Supplementary Information:**

The online version contains supplementary material available at 10.1007/s00216-026-06504-3.

## Introduction

Steroid hormones are essential for the optimal function of the human body, as they can regulate gene transcription, interact with DNA, and impact signaling pathways through cell-specific receptors. They play significant physiological roles in metabolic processes and can be instrumental to the diagnosis, treatment, and prevention of a variety of diseases and disorders. Despite their importance, the profile of steroid hormones circulating in the body, as well as their delicate equilibrium and the factors that can disturb this balance, remains largely unknown.

Steroid hormones are synthesized from cholesterol through a series of highly regulated enzymatic steps including hydroxylation, reduction, methylation, and demethylation. In circulation, these hormones exist in an equilibrium, and disruptions to their synthesis or metabolism can lead to various diseases and disorders [1, 2]. For instance, a deficiency in the 5α-reductase enzyme interrupts the conversion of testosterone to dihydrotestosterone, negatively affecting male sexual development [3]. Additionally, in cancer biology, understanding the context-dependent effects of hormones, such as estradiol, progesterone, and testosterone, on cancer risk and progression, is vital [4–6]. A detailed steroid hormone profile would enhance diagnostic and therapeutic strategies, particularly in hormone-driven cancers [4, 7]. Moreover, the measurement of circulating estrogens, androgens, progestogens, and their sulfated precursors as a panel may yield new insights into the impacts of endocrine-disrupting chemicals on the endocrine system; these chemicals function by inhibiting receptors and interfering with hormone synthesis. This hormone profiling approach aligns with the principles of personalized medicine, providing unique mechanistic insights for various diseases, thus allowing for tailored treatment strategies to address patient-specific biochemical characteristics, which may help with the assessment of diseases, such as certain cancers, bone and cardiovascular diseases, and disorders of sexual development [8, 9].

Traditional steroid hormone assays used in patient care typically measure individual hormones, necessitating the use of multiple individual assays to generate a comprehensive steroid hormone panel profile, which can be resource-intensive [10, 11]. Previous measurements were often conducted in singlicate for individual hormones, employing various techniques from immunoassay to mass spectrometry measurement approaches. This approach has led to data normalization based on assumptions specific to each technique, which can compromise the reliability of statistical analyses. Consequently, these adjustments have increased the total allowable error, ultimately diminishing the credibility of the results [12–14]. Additional concerns have been raised regarding the accuracy, sensitivity, and reliability of conventional immunoassays, particularly for samples with low levels of circulating steroids [15–17]. Several published high-performance liquid chromatography coupled with mass spectrometry (HPLC-MS/MS) methods quantify steroid hormones in both individual hormone and panel formats; however, many published methods involve derivatization or hydroxylation of steroid hormones, measuring the fragments of the derivatized compounds rather than the actual analytes, which is reported to decrease assay selectivity [18–21]. Other methods may require high sample volumes or use harmful solvents to extract steroids from human specimens [22, 23]. Furthermore, some panel methods lack the sensitivity needed to measure analytes at concentrations clinically relevant to specific population subgroups, such as postmenopausal women with limits of quantification that are an order of magnitude higher than what is required for those subgroups [24, 25].

While LC-MS/MS multi-analyte methods for steroids have advanced significantly, they still face limitations in achieving the necessary sensitivity for low abundance analytes, such as estrogens [21, 26–28]. Current analytical methods for steroid hormone panels focus on steroid hormones with certain polarity and do not cover polar as well as non-polar steroid hormones. Additionally, selectivity for compounds with multiple isomers and the differentiation between endogenous and exogenous analytes can be challenging, decreasing the effectiveness of hormone measurements across diverse populations. Recent publications on LC-MS/MS methods for some of these analytes indicate that they are not as comprehensive or sensitive as required, making them unsuitable for measurements in all populations [21, 26–28]. Consequently, there is a critical need for new analytical methods that can simultaneously measure multiple androgens, estrogens, and progestogens with high specificity and sensitivity.

Steroid hormones can be found in both free and protein-bound forms in circulation with free hormones representing on average 1–3% of circulating hormone levels [29]. Free hormones can either be measured directly, for example, after equilibrium dialysis, or calculated using total hormone concentrations and binding protein concentrations (SHBG and albumin) often utilizing equilibrium dialysis approaches, which are labor-intensive and difficult to standardize [29–31]. When needed, free hormone concentrations can be estimated from total hormone levels and binding protein concentrations. For example, free testosterone levels are determined using total testosterone and SHBG concentrations [32]. The measurement of total hormones most closely aligns with standard clinical practice, as most reference ranges and diagnostic thresholds are based on total hormone levels. Additionally, total hormone measurements are more commonly used for population studies.

Herein, we describe a novel isotope-dilution high-performance liquid chromatography mass spectrometry (HPLC-MS/MS) method for the simultaneous direct measurement (non-derivatized) of a broad range of hormones, including both non-polar and polar steroids, in a single panel. More specifically, this method quantifies eight steroid hormones in serum, including estradiol (E2), estrone (E1), estrone sulfate (E1S), total testosterone (TT), androstenedione (AD), dehydroepiandrosterone sulfate (DHEAS), progesterone (P4), and 17-hydroxyprogesterone (17-OHP). This method is both highly sensitive and selective, measuring total hormone concentrations, and can be used to measure steroid hormones in the general population, as well as for standard diagnostic workflows, thus providing a practical and reproducible tool for both epidemiological research and clinical use.

## Materials and methods

### Materials and chemicals

Certified primary reference materials and stable isotope-labeled controls corresponding to each steroid hormone measured by the method (Supplemental Table 1) were purchased from the National Measurement Institute, Australia (NMI), National Metrology Institute, Japan (NMIJ), Cerilliant Corporation, (Round Rock, TX), IsoSciences (King of Prussia, PA), and Cambridge Isotope Laboratories, Inc. (Andover, MA). Additional steroidal compounds used for interference testing were obtained from Cerilliant, NMI, and Sigma-Aldrich (St. Louis, MO) (Supplemental Table 2). Reagents including ethanol, methanol, and ammonium fluoride were of analytical grade and acquired from Thermo Fisher Scientific (Hanover Park, IL). Synthetic serum for the preparation of the quality control samples was procured from UTAK Laboratories (Valencia, CA).

Three levels of serum-based reference material for E2 (BCR 576: 114 pmol/L, BCR 577: 690 pmol/L, and BCR 578: 1340 pmol/L) were purchased from the European Union Joint Research Center (EU-JRC, Geel, Belgium). Two levels of serum-based standard reference materials (SRM) for TT (SRM 971M: 22.3 nmol/L and SRM 971F: 0.96 nmol/L) and one SRM level for P4 (SRM 971F: 6.20 nmol/L) were obtained from the National Institute for Standards and Technology (NIST, Gaithersburg, MD).

### Individual donor serum samples

Deidentified human sera from 200 individual adult donors were commercially sourced from BioIVT (Westbury, NY) and consisted of 100 males and 100 females, with 51 premenopausal females and 49 postmenopausal females. For this work, postmenopausal status was defined as age equal to or greater than 60 years, while premenopausal status was defined as age less than 50 years, based on the reported average age of menopause onset [33, 34]. Additionally, 68 deidentified individual adolescent girl samples acquired from the National Institute of Environmental Health Sciences (NIEHS) were used to assess the method. For BioIVT samples, local Institutional Review Boards (IRBs) approved sample collection protocols. The samples from adolescent girls were obtained from NIEHS, as described previously, and the study was reviewed and approved by the NIEHS IRB [35]. Signed informed assent and consent were obtained by NIEHS from each subject and her parent, respectively. Documentation for all samples was provided to the CDC, where it was reviewed and approved by the CDC Human Subjects Coordinator

Deidentified individual donor sera from 89 donors were obtained from the CDC Hormones Standardization Program (HoSt) for TT and E2. Materials used in the CDC HoSt Program are commercially sourced, unaltered human sera from single donors obtained following the CLSI C37-A protocol [36, 37]. The reference measurements were performed in the CDC Reference Laboratory, and the concentrations were assigned by the CDC LC-MS/MS Reference Methods for TT and E2 [38, 39]. Research involving human subjects complied with all relevant national regulations and institutional policies and is in accordance with the tenets of the Helsinki Declaration (as revised in 2013).

### Calibrators, internal standards, and quality control samples

The primary calibrator solutions for the individual steroids were prepared by dissolving the certified reference materials in anhydrous ethanol (EtOH). Intermediate calibrator solutions were created by combining each of the primary solutions and diluting the resulting mixture in EtOH. Ten levels of calibrator working solutions were prepared by combining various volumes of the intermediate calibrator solutions in a 20/80 EtOH/water v/v mixture (Supplemental Tables 3A and 3B). Individual isotopically labeled standards were diluted in EtOH and combined to create an internal standard (IS) working solution (Supplemental Table 4).

Quality control (QC) samples were prepared at low, medium, and high steroid hormone concentrations to span the measurement range typically observed in the general population. QCs were prepared by adding intermediate calibrator solutions to synthetic serum and mixing for 60 min. The QC samples were characterized by measuring them in duplicate over a period of 51 days, with acceptance criteria defined using previously described statistical procedures [40].

### Sample preparation

All sample processing steps are performed using a robotic liquid handler (Hamilton Microlab STARLet Liquid Handler, Reno, NV) equipped with a 96-channel head and an 8-channel individual pipette head. Each plate run is comprised of 73 serum samples, 10 calibrators, 6 QC samples, and 7 saline or reagent blanks. The calibrators and QC samples are processed and analyzed in the same run and in the same manner as patient specimens. Sample preparation is performed using a stepwise liquid-liquid extraction procedure using solvents with different polarities. In brief, 100 µL of the IS working solution is added to 200 µL of sample and mixed for 45 min at room temperature. Then, 100 µL of 0.5 mol/L ammonium acetate buffer (pH 5.5) is added to each sample 30 min before mixing. Steroids are first extracted with ethyl acetate, followed by subsequent extractions using 70/30 (v/v) ethyl acetate/hexane and 1-butanol. Each extraction is performed for 15 min with 500 µL of extraction solvent. Each extract is mixed with 200 µL of ammonium bicarbonate (0.2 mol/L) at pH 8.0. All extracts are then combined, dried using nitrogen, and reconstituted in 150 µL of 30/70 (v/v) methanol/water mixture for UHPLC-MS/MS analysis.

### UHPLC-MS/MS

UHPLC-MS/MS analysis is conducted using a triple quadrupole mass spectrometer (Sciex 6500, Foster City, CA) coupled to a UHPLC system (Shimadzu Nexera, Columbia, MD). The UHPLC system is equipped with a reverse-phase column (Accucore Phenyl Hexyl, 2.6 µm particle size, 150 x 3 mm), which is connected to a C13 guard column (4 x 2 mm, Phenomenex, Torrance, CA). Steroid hormones are eluted using two solvents: eluent A, which consists of a 30/70 (v/v) methanol/water mixture with 0.6 mmol/L ammonium fluoride, and eluent B, which is pure methanol. A stepwise gradient is applied as follows: 0–1 min at 16% B, 1–7 min from 16 to 56% B, and 7–17 min from 56 to 74% B at a flow rate of 250 µL/min. The injection volume is 50 µL. Detection and quantification are performed using selected reaction monitoring in both positive and negative ionization modes. The following instrument settings are used: source temperature of 650 °C; curtain gas at 45; collision gas at 9; ion source gas 1 at 55 psi; ion source gas 2 at 70 psi; and ion spray voltage at −4500/4500 kV. Mass spectrometer settings, including quantifier and qualifier *m/z* values, corresponding collision energy, and declustering potential were optimized for each compound (Supplemental Table 5).

### Data analysis

The UHPLC-MS/MS results were processed using MultiQuant software (Sciex, Foster City, CA). Weighted calibration curves were generated by plotting the peak area ratios for each calibrator against the corresponding internal standard, normalized to the inverse of the target concentrations (1/[target concentrations]). Statistical analyses were performed using SAS/STAT software version 9.2 (SAS Institute, Inc., Cary, NC) and Analyze-It Software, Ltd., version 4.65.2 (Leeds, UK).

### Accuracy

The accuracy of the measurements for TT, E2, and P4 was determined by comparing the results obtained with this method to target values assigned to certified reference materials (CRM) as outlined in NIST Special Publication 829 [41]. Additionally, for TT and E2, comparisons to values obtained by a reference measurement procedure were performed using 89 single-donor sera samples acquired from the CDC Hormone Standardization Program, as described above, and employing Deming regression and bias plot analyses in accordance with CLSI guideline EP09c [42]. For the other analytes, accuracy was assessed through spike-recovery analysis, which involved comparing the expected spiked concentration to the measured concentration of the spiked samples after subtraction of endogenous steroid concentrations measured in the base serum sample prior to spiking. The spiking analysis was performed in triplicate over two days (*n* = 6) using two different serum samples spiked at three different concentrations.

### Precision

Three levels of QC materials were analyzed over 51 days (with 2 runs per day and 2 replicates per run) over a one-year period. The data were analyzed as described previously, using a one-way ANOVA to determine within-day imprecision (repeatability), between-day imprecision, and total imprecision [40].

### Linearity

Linearity and the most appropriate weighting were determined by assessing the average sum of squared residuals (ASSR) and the average relative sum of squared residuals (RASSR) from 20 calibration curves, each run independently over 20 days. The best-fitting regression model for linear and polynomial forms with no weighting and weights of 1/X, 1/X^2^, or 1/(variance of Y) was evaluated. The ASSR was calculated by summing the squared differences between the predicted and observed Y values of the models. The RASSR was calculated by dividing the ASSR by the average of the Y values. A statistical test for lack of fit of the linear calibration curves, along with the smallest ASSR and second-smallest RASSR among all models, indicated that a weight of 1/X was the best fit for the calibration curves of each analyte. The evaluation of linearity was performed as outlined in CLSI EP06 [43].

### Limit of detection (LOD) and upper limit of quantification (ULOQ)

The LOD was determined by measuring the five lowest calibrators over 5 days and estimating the standard deviation (SD) at zero concentration (SD_0_) using the method described by Taylor [44]. In brief, the SD of the results was plotted against the concentration, and a regression line was generated to estimate SD_0_. The LOD was defined as 3*SD_0_. The lowest calibrator concentration was used as the lower limit of quantitation (LLOQ), where we achieved less than 10% imprecision for all analytes except DHEAS, which had an imprecision of 12% [45].

A sample dilution experiment was conducted by diluting a single-donor sample with a saline solution or synthetic serum up to 10-fold in triplicate and comparing the diluted sample concentration measurements against the undiluted sample concentration. Dilutions with a recovery of 100 ±10% were considered acceptable [46]. The extent to which the diluted sample could be measured accurately was used to calculate the upper limit of quantitation (ULOQ).

### Selectivity

Analytical selectivity was assessed by comparing the retention times and peak resolutions of 27 structurally analogous steroid hormones and other potentially interfering substances with relative molecular masses similar to the analytes measured in the steroid hormone panel method. The absence of peaks with the same mass transitions as the analytes or IS at the relevant retention times confirmed that the structural analogues did not interfere with the quantification of the eight steroids included in the panel. Analyte-free, natural serum cannot be obtained; however, reagent blank samples did not show any peaks at the same retention time as the eight steroids measured. Additionally, since the method does not utilize derivatization, the specific mass transitions for each analyte allow the use of quantification-ion/confirmation-ion (QI/CI) ratios to identify potential interferences. The QI/CI ratios of 268 single-donor serum samples were compared to those of neat calibrator solutions. A difference greater than ±20% of the target ratio established using the neat solutions was used to determine the presence of an interfering compound [47].

### Evaluation of matrix effects

The effect of different sample matrices was determined following procedures as previously described [38, 39, 48]. Five sample matrices, including synthetic serum, 0.9% saline solution, charcoal-stripped serum, individual donor male serum, and individual donor female serum, and one set of neat samples in 20/80 (v/v) EtOH/water mixture were evaluated. A 6-point calibration curve ranging from the third to eighth calibrators was prepared in each matrix. All matrix-type samples were subjected to the sample preparation described above. The MS response (area count ratio of analyte to IS) was compared across all five matrices to the MS response of the neat samples. The sample matrix effect (ME) was calculated with the following equation: $ME\%=\left(\frac{B}{A}\right)*100$, where B is the area count ratio of analyte to IS obtained from samples in matrix, and A is the area count ratios in matrix-free samples. The slopes derived from all calibration curves for all matrices were compared to that of the slope in the neat samples. A difference greater than ± 20% of the target recovery was used to determine if there were any matrix effects.

### Measurement uncertainty

Expanded uncertainty was calculated according to the ISO Guide on the Expression of Uncertainty in Measurement 2008 [49]. The estimated variance of Type A uncertainty was obtained from the imprecision of the repeated measurements, while the Type B uncertainty budget was derived from uncertainties at the concentrations of the certified reference materials, the uncertainty of the purity of the reference material (when applicable), and the inaccuracy associated with the use of pipettes and volumetric flasks. Type A and B uncertainties were combined quadratically to calculate the standard uncertainty. The expanded measurement uncertainty (U) at the 95.45% level of confidence can be calculated by multiplying the standard uncertainty by a coverage factor of 2. Low, medium, and high QC materials were used for uncertainty assessments. The desired and minimal standard relative measurement uncertainty for each of the analytes were determined based on biological variation data or using the FDA guidelines when biological variation information was not available (Supplemental Table 6) [50–53]. 

### Method application

To assess the applicability of this steroid hormone panel method for measuring eight steroid hormones in the general population, 268 serum samples from adult males and females and adolescent girls were analyzed [54]. This included samples from postmenopausal women, known to have steroid hormone concentrations that are challenging to some commercial hormone assays. Applicability to younger children is inferred from analytical performance and was not directly validated for this manuscript.

## Results and discussion

**Fig. 1:**
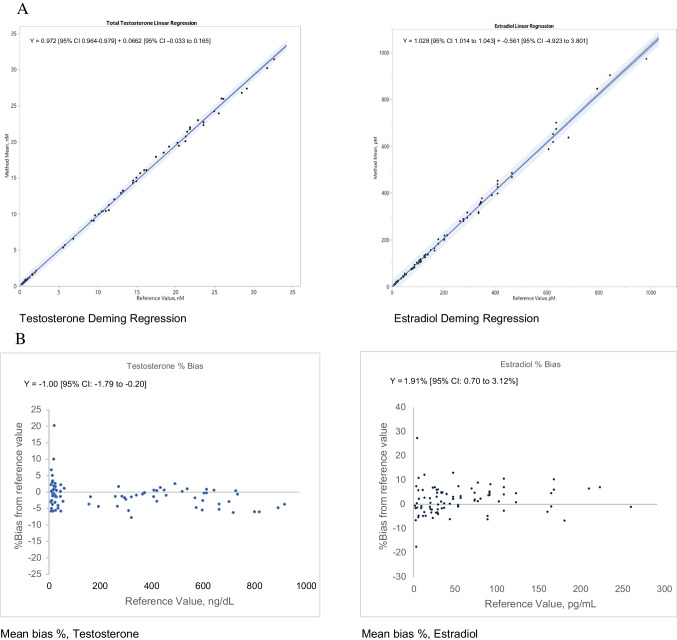
Comparison of results between the steroid hormone panel method and the E2 and TT reference methods using Deming regression and bias plot analyses.** A** Comparisons between the steroid hormone panel method and reference method were conducted by comparing measured values to reference method values for 89 secondary reference materials obtained from CDC HoSt Program for TT and E2. The steroid hormone panel method showed very high agreement (solid blue line) with the reference method, as demonstrated by narrow confidence intervals (light blue solid). Deming regression slopes and intercepts are provided as insets in each figure panel. **B** Difference in bias plots comparing the steroid hormone panel method to the reference method for the same 89 secondary reference materials showed very high agreement for TT and E2

**Fig. 2:**
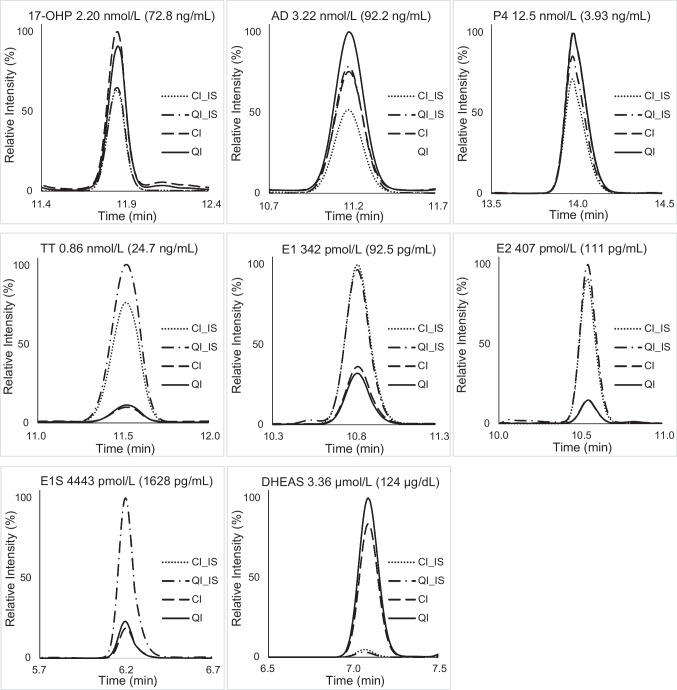
Representative LC-MS/MS chromatograms of steroid hormone analytes and stable isotope-labeled internal standards in a female donor serum sample. Chromatograms for each analyte show quantification and confirmation transitions with bell-shaped peaks, targeted retention times, and high signal-to-noise ratios

The newly developed LC-MS/MS steroid hormone panel method described in this manuscript is capable of simultaneously quantifying eight clinically relevant steroid hormones and meets the requirements described in CLSI C62 [47]. The method is highly accurate and precise and meets or exceeds suggested analytical performance requirements (Supplemental Table 6). The average bias for E2 as compared to metrologically certified serum-based reference materials from EU-JRC was −0.3%. The biases for TT and P4 as compared to SRMs from NIST were 1.35% and 1.9%, respectively (Table 1). Measurements from the steroid hormone panel method showed a very high level of agreement compared to TT and E2 reference values assigned by CDC Reference Laboratories for 89 secondary reference material samples obtained from the CDC HoSt Program, with narrow confidence intervals (Figure 1). For TT, the regression analysis yielded a slope of 0.972 [95% CI: 0.964 to 0.979] and an intercept of 0.066 [95% CI: −0.033 to 0.165] (Fig. 1A). For E2, the slope was 1.028 [95% CI: 1.014 to 1.043] with an intercept −0.561 [95% CI: −4.923 to 3.801] (Figure 1A). The mean bias for TT was −1.00 [95% CI: −1.79 to −0.20], while the mean bias for E2 was 1.91 [95% CI: 0.70 to 3.12] (Figure 1B). Spike-recovery experiments were conducted using well-characterized, commercially sourced CRMs to assess accuracy for the analytes for which no CRMs were available from metrological institutes. The spike-recovery results demonstrated that the average recovery for the analytes assessed ranged between 92.1% and 107.1% (Table 2). The observed biases were within the limits suggested for bioanalytical methods [53], as used in CDC’s Clinical Standardization Programs for TT and E2 [37, 38, 42, 55–57], and were consistent with the desirable biological variability [51, 52] (Supplemental Table 6). The high degree of accuracy for this method was achieved by using certified reference materials as calibrators (Supplemental Table 1), when available, and using stable isotope-labeled internal standards. Individual, representative chromatograms for each analyte and its corresponding IS for a female donor sample are shown in Fig. 2. The average retention time was determined for each analyte across 20 independent runs and were within ± 2.5% of the target retention time, with all peaks showing appropriate chromatographic separation and resolution [47].

**Table 1:** Method biases relative to certified reference materials. The measured values for progesterone (P4), total testosterone (TT), and estradiol (E2) samples were compared to the values assigned to each certified reference material sample. Each sample tested is listed with its analyte, reference values, and measured values in SI Units and conventional units with 95% confidence intervals, as well as bias percentages with 95% confidence intervals

Sample (analyte)	Reference value	Measured value (95% CI)	Bias in % (95% CI)
SRM 971 F (P4)	6.20 nmol/L	6.32 nmol/L (6.27–6.37)	1.9
	1.95 ng/mL	1.99 ng/mL (1.97–2.00)	(1.1 to 2.7)
SRM 971 F (TT)	0.96 nmol/L	1.00 nmol/L (0.98–1.03)	4.8
	27.7 ng/dL	29.0 ng/dL (28.3–29.8)	(2.1 to 7.6)
SRM 971 M (TT)	22.3 nmol/L	21.8 nmol/L (21.5–22.2)	−2.1
	643 ng/dL	630 ng/dL (619–640)	(−.7 to −0.5)
BCR 576 (E2)	114 pmol/L	114 pmol/L (111–116)	−0.2
	31.1 pg/mL	31.0 pg/mL (30.3–31.7)	(−2.5 to 2.2)
BCR 577 (E2)	690 pmol/L	686 pmol/L (670–701)	−0.6
	188 pg/mL	187 pg/mL (182–191)	(−3.0 to 1.7)
BCR 578 (E2)	1340 pmol/L	1338 pmol/L (1312–1364)	−0.1
	365 pg/mL	365 pg/mL (357–372)	(−2.1 to 1.8)

**Table 2:** Spike-recovery analysis of steroid hormones without metrologically available CRMs. Method accuracy, expressed as percent recovery, was evaluated by spiking two base samples at three different concentrations prior to measurement by the steroid hormone panel method in triplicate over 2 days (*n* = 6). Endogenous concentrations of the base samples were determined prior to spiking

	Spike solution	17-OHP	AD	E1	E1S	DHEAS
Measured concentration nmol/L (ng/dL)						
Sample 1	Endogenous	0.87 (28.8)	1.30 (37.3)	126 (34.0)	498 (182)	1.50 (55.2)
Sample 1	Spike 1	1.44 (47.5)	2.07 (59.4)	208 (56.2)	680 (249)	1.84 (67.8)
Sample 1	Spike 2	1.98 (65.3)	2.90 (83.0)	279 (75.4)	852 (312)	2.16 (79.7)
Sample 1	Spike 3	2.49 (82.4)	3.75 (107)	378 (102)	1076 (394)	2.49 (91.8)
Sample 2	Endogenous	3.82 (126)	5.70 (163)	548 (148)	1540 (564)	3.91 (144)
Sample 2	Spike 1	5.21 (172)	7.82 (224)	786 (212)	2084 (764)	4.99 (184)
Sample 2	Spike 2	6.63 (219)	9.54 (273)	1010 (273)	2580 (945)	6.12 (226)
Sample 2	Spike 3	8.62 (285)	12.1 (348)	1355 (366)	3506 (1284)	7.74 (285)
Anticipated concentration nmol/L (ng/dL)						
Sample 1	Spike 1	1.33 (43.8)	1.96 (56.3)	198 (53.5)	732 (268)	1.70 (62.8)
Sample 1	Spike 2	1.86 (61.6)	2.65 (76.0)	285 (77.0)	942 (345)	2.06 (76.1)
Sample 1	Spike 3	2.40 (79.4)	3.36 (96.3)	352 (95.1)	1094 (401)	2.38 (88.0)
Sample 2	Spike 1	5.23 (173)	7.21 (207)	707 (191)	2336 (856)	5.26 (194)
Sample 2	Spike 2	6.59 (218)	9.71 (278)	936 (253)	3028 (1109)	6.33 (233)
Sample 2	Spike 3	8.35 (276)	12.3 (352)	1199 (324)	3628 (1329)	7.69 (284)
Recovery (%)						
Sample 1	Spike 1	108.3	105.5	105.1	92.9	108.0
Sample 1	Spike 2	106.0	109.3	97.9	90.5	104.7
Sample 1	Spike 3	103.7	111.5	107.6	98.3	104.3
Sample 2	Spike 1	99.7	108.5	111.1	89.2	95.0
Sample 2	Spike 2	100.6	98.2	107.9	85.2	96.7
Sample 2	Spike 3	103.2	99.0	113.1	96.6	100.6
Overall mean		**103.6**	**105.3**	**107.1**	**92.1**	**101.5**

The steroid hormone panel method demonstrates high precision across all eight hormones measured at three concentration levels, with total imprecision ranging from 3.5 to 10.7% CV (Table 3). Mean analyte concentrations were calculated from 204 replicates (*n* = 204) and are reported in both SI and conventional units. Within-day imprecision was determined from four replicates (*n* = 4), between-day imprecision from 51 replicates (*n* = 51), and total imprecision from all replicates (*n* = 204). Total imprecision values for all analytes fell within the recommended limits for bioanalytical methods [51, 53], and those suggested based on biological variability data [51, 52] (Supplemental Table 6). The consistent high level of precision was achieved using automated pipetting instruments and the inclusion of calibration curves with each run to ensure consistency across days. Further improvements in measurement consistency were achieved by eliminating a protein precipitation step commonly used in sample preparation by other analytical methods and equilibrating the internal standards prior to sample processing, as described previously [58].

**Table 3:** Assay precision for eight steroid hormones measured in high, medium, and low concentration quality control (QC) samples. Data are reported as mean concentrations (*n* = 204) with both SI and conventional units. Within-day imprecision was determined from four replicates (*n* = 4), between-day imprecision from 51 replicates (*n *= 51), and total imprecision from all replicates (*n* = 204). Imprecision is expressed as coefficient of variation (%CV)

Sample description	Mean concentration (*n* = 204)							
	17-OHP nmol/L (ng/dL)	AD nmol/L (ng/dL)	P4 nmol/L (ng/mL)	TT nmol/L (ng/dL)	E1 pmol/L (pg/mL)	E2 pmol/L (pg/mL)	E1S pmol/L (pg/mL)	DHEAS µmol/L (µg/dL)
QC High	3.69 (122)	9.70 (278)	23.8 (7.50)	20.9 (603)	1715 (464)	3111 (847)	1696 (621)	3.22 (119)
QC Medium	1.25 (41.3)	3.01 (86.1)	7.67 (2.41)	7.07 (204)	621 (168)	990 (270)	568 (208)	1.20 (44.4)
QC Low	0.26 (8.64)	0.56 (16.1)	1.47 (0.46)	1.35 (38.9)	119 (32.3)	192 (52.6)	105 (38.4)	0.23 (8.40)
	Within-day imprecision (%CV, *n* = 4)							
	**17-OHP**	**AD**	**P4**	**TT**	**E1**	**E2**	**E1S**	**DHEAS**
QC High	4.7	3.7	4.5	3.2	4.2	4.8	5.9	6.1
QC Medium	5.3	3.3	4.7	3.3	5.3	5.2	6.8	6.5
QC Low	7.2	4.1	5.7	4.1	7.7	6.0	7.8	6.9
	Between-day imprecision (%CV, *n* = 51)							
	**17-OHP**	**AD**	**P4**	**TT**	**E1**	**E2**	**E1S**	**DHEAS**
QC High	2.0	1.8	1.8	2.0	0.5	2.7	7.1	3.4
QC Medium	1.2	1.1	2.2	1.7	2.6	1.0	7.0	2.3
QC Low	5.3	2.2	3.2	2.5	0.5	1.4	2.8	8.1
	Total imprecision (%CV, *n* = 204)							
	**17-OHP**	**AD**	**P4**	**TT**	**E1**	**E2**	**E1S**	**DHEAS**
QC High	5.1	4.1	4.8	3.7	4.2	5.5	9.3	7.0
QC Medium	5.4	3.5	5.1	3.7	5.9	5.3	9.8	6.9
QC Low	9.0	4.6	6.5	4.8	7.7	6.5	8.3	10.7

The described method demonstrates high analytical sensitivity, enabling quantification of target analytes across the broad concentration ranges observed in men, pre- and postmenopausal women, and children. LODs ranged from 4.81 pmol/L for E1 to 6000 pmol/L for DHEAS (Table 4). The established LODs are comparable to or lower than those reported for alternative analytical approaches [26, 59–66]. While derivatization was shown to further decrease LOD concentrations for certain analytes [67], its application can be restricted by analyte-specific compatibility [68] and, in some cases, may compromise selectivity when only the fragments of the derivatized compounds are monitored [69, 70]. Samples from children and postmenopausal women often exhibit lower steroid hormone concentrations [71]. Of the 117 postmenopausal and adolescent female samples tested with this method, only ~2% of results fell below the lower reportable limit. To establish the upper limits of the extended analytical reportable range for each analyte in the method, multiple dilutions were assessed for accuracy. For most analytes, dilutions of up to 10-fold with saline were acceptable, whereas DHEAS tolerated dilutions of up to 7-fold (Table 5). Taking dilution into consideration, the method achieved reportable ranges spanning 3 to 4 orders of magnitude for all analytes. Of the 268 individual serum samples analyzed, only 2 exceeded the upper reportable range.

**Table 4:** Limits of detection (LOD) and quantitation (LOQ) and reportable ranges for eight steroid hormones. The LOD, LOQ, and reportable ranges for each of the measurands in the steroid hormone panel method demonstrate high sensitivity and broad dynamic ranges that span 3 orders of magnitude across samples from men, women, and children

	Analytes							
	17-OHP nmol/L (ng/dL)	AD nmol/L (ng/dL)	P4 nmol/L (ng/mL)	TT nmol/L (ng/dL)	E1 pmol/L (pg/mL)	E2 pmol/L (pg/mL)	E1S pmol/L (pg/mL)	DHEAS µmol/L (µg/dL)
LOD	0.012 (0.41)	0.029 (0.82)	0.027 (0.009)	0.020 (0.57)	4.81 (1.30)	6.31 (1.72)	5.57 (2.04)	0.006 (0.22)
LOQ	0.024 (0.79)	0.033 (0.95)	0.156 (0.05)	0.080 (2.32)	13.7 (3.70)	9.66 (2.63)	10.8 (3.96)	0.077 (2.83)
Reportable range (LLOQ–ULOQ*)	0.024 (0.79)–131 (4323)	0.033 (0.95)–183 (5250)	0.156 (0.05)–622 (1957)	0.080 (2.32)–443 (12,783)	13.7 (3.70)–20,195 (5460)	9.66 (2.63)–62,658 (17,067)	10.8 (3.96)–69,560 (25,480)	0.021 (0.76)–78.8 (2906)

*LLOQ* lower limit of quantitation, *ULOQ* upper limit of quantitation

**Table 5:** Recovery of steroid hormones after serum dilution. Samples were diluted with saline and accuracy by spike recovery ranged from ~ 99 to 103% for most analytes with the dilution factors indicated

Analyte	Dilution factor	Recovery (%, SD)^a^
17-OHP nmol/L (ng/dL)	10	99.3 (3.1)
AD nmol/L (ng/dL)	10	99.7 (3.6)
P4 nmol/L (ng/dL)	10	98.6 (3.2)
TT nmol/L (ng/dL)	10	100.8 (3.2)
E1 nmol/L (ng/dL)	10	98.8 (6.1)
E2 pmol/L (pg/mL)	10	101.5 (6.5)
E1S pmol/L (pg/mL)	10	98.6 (4.6)
DHEAS µmol/L (µg/dL)	7	102.9 (4.4)

^a^Average recovery calculated from 3 replicate measurements

High analytical selectivity was achieved by ensuring that potentially interfering compounds were either chromatographically resolved or not detected owing to the selection of mass transitions specific to each analyte to be quantified. This was accomplished through monitoring analyte-specific fragmentation patterns and using QI/CI ratios to detect unknown interferences. A total of 27 compounds frequently found in circulation, that were isomers, or that were structurally analogous to the target hormones of the method were evaluated as potential interfering compounds. No co-elutions or mass-to-charge interferences were observed (Supplemental Table 2, Supplemental Figure 1). Among the 268 individual donor samples, fewer than 6% were flagged as being outside the allowable QI/CI ratio limits. Deviations from the calibrators’ QI/CI ratio range were primarily attributable to insufficient CI intensity, which precluded accurate ratio determination (Table 6). For the majority of the samples, QI/CI ratios remained well within the recommended ±20% range relative to calibrators [47]. The use of stable isotope-labeled internal standards for each analyte allowed for effective corrections for analyte loss during sample preparation and LC-MS/MS analysis. Moreover, analysis of IS-only samples revealed no additional peaks in the analyte signal, confirming the absence of isotopic interferences associated with the selected internal standards.

**Table 6:** Selectivity assessment based on QI/CI ratios. A total of 268 individual donor serum samples (100 males, 51 premenopausal females < 50 years, 49 postmenopausal females > 60 years, and 68 girls) were analyzed. For samples with QI/CI ratios falling within the reportable range, the ratios were compared to the calibrator QI/CI ratios (*n* = 60) to evaluate if they were within ± 20% of the calibrator values. For each analyte, the percentage of individual donor samples with QI/CI ratios falling outside this range is reported. Deviations from calibrator ranges were primarily attributable to insufficient CI intensity, which precluded accurate ratio determination. Most samples remained within the recommended ± 20% of calibrator ratios, confirming the robustness of the assay selectivity

Analyte	Calibrator QI/CI (mean)/calibrator QI/CI (range)	Samples outside of the ± 20% QI/CI calibrator ranges (%)
17-OHP (*n* = 266)	0.93 (0.75–1.12)	0.37
AD (*n* = 267)	1.37 (1.09–1.64)	0.37
P4 (*n* = 220)	1.11 (0.89–1.34)	1.87
TT (*n* = 268)	1.22 (0.98–1.47)	0.37
E1 (*n* = 268)	2.13 (1.71–2.56)	2.99
E2 (*n* = 233)	1.01 (0.81–1.21)	5.97
E1S (*n* = 266)	4.29 (3.43–5.14)	1.87
DHEAS (*n* = 266)	1.44 (1.15–1.73)	4.48

^*^n, number of samples that fell within reportable range

The steroid hormone panel method demonstrated minimal susceptibility to matrix effects. To evaluate this, mean matrix effect percentages (ME%), calibration curve slopes, and correlation coefficients were calculated for 5 different matrices, which included synthetic serum, 0.9% saline, charcoal-stripped serum, and male and female individual donor serums (Table 7). Mean ME% values ranged from 94.4 to 104%. Calibration curve slopes and correlation coefficients were consistent across all matrices, confirming method robustness. Despite the unique physiochemical properties of the matrices evaluated, sequential liquid-liquid extractions with solvents of different polarities ensured effective recovery of all analytes across matrices. Additionally, the solvents used in this method are less volatile than those used by other published methods [20, 54, 68, 72, 73], enabling reliable pipetting by the automated liquid handling system.

**Table 7:** Matrix effect (ME%), calibration curve slopes, and correlation coefficients (R^2^) for steroid hormone quantitation across different matrices. Mean ME% values were between 94 and 104%, where all ME% were > 92%. Calibration curve slopes were consistent between neat solutions and biological matrices. R^2^ values exceeded 0.99 for all analytes assessed, demonstrating excellent linearity across all matrices evaluated

Matrix description	ME%							
	17-OHP	AD	P4	TT	E1	E2	E1S	DHEAS
Synthetic serum	98.2	97.4	92.9	97.3	101	106	91.9	102
0.9% saline	102	98.7	93.8	101	99.6	103	103	108
Charcoal stripped serum	102	93.8	96.7	98.0	101	106	95.3	97.8
Male serum (individual donor)	102	99.1	92.6	104	101	104	101	97.3
Female serum (individual donor)	103	100	95.8	98.8	103	101	101	97.6
Mean ME% (*n* = 18) **(95% CI)**	**101** **(99.1–103)**	**97.9** **(95.5–100)**	**94.4** **(91.9–96.9)**	**99.9** **(97.8–102)**	**101** **(99.9–102)**	**104** **(103–105)**	**98.2** **(95.9–101)**	**100** **(98.2–103)**
	**Calibration curve slopes**							
	**17-OHP**	**AD**	**P4**	**TT**	**E1**	**E2**	**E1S**	**DHEAS**
Neat (20% ethanol in water)	0.0204	0.0155	0.0036	0.0052	0.0414	0.0424	0.0234	1.8545
Synthetic serum	0.0215	0.0155	0.0036	0.0049	0.0423	0.0434	0.0213	1.9533
0.9% Saline	0.0218	0.0157	0.0038	0.0051	0.0418	0.0427	0.0239	2.0354
Charcoal stripped serum	0.0218	0.0152	0.0036	0.0051	0.0425	0.0438	0.0216	1.9663
Male serum (individual donor)	0.0212	0.0146	0.0035	0.0053	0.0423	0.0435	0.0219	1.7119
Female serum (individual donor)	0.0201	0.0147	0.0035	0.0048	0.0426	0.0434	0.0219	1.6699
	**R**^**2**^							
	**17-OHP**	**AD**	**P4**	**TT**	**E1**	**E2**	**E1S**	**DHEAS**
Neat (20% ethanol in water)	1.0000	0.9999	0.9998	0.9999	0.9996	0.9999	0.9994	0.9971
Synthetic serum	0.9998	0.9999	0.9999	0.9994	0.9998	0.9996	0.9994	0.9989
0.9% saline	0.9985	0.9997	0.9987	0.9997	0.9997	0.9998	0.9997	0.9978
Charcoal stripped serum	0.9996	0.9998	0.9993	0.9990	0.9995	0.9986	0.9997	0.9993
Male serum (individual donor)	0.9993	0.9998	0.9977	0.9994	0.9990	1.0000	0.9980	0.9949
Female serum (individual donor)	0.9994	0.9998	0.9998	0.9958	0.9989	0.9996	0.9996	0.9965

The calculated standard measurement uncertainties for 17-OHP, AD, P4, TT, E1, E2, E1S, and DHEAS are all within the desirable analytical specifications defined for each analyte (Supplemental Table 7). Across the concentration range evaluated, measurement uncertainties for 7 out of 8 analytes are lowest for the medium and high QC levels, with values ranging from 3.5 to 9.8%, whereas E1S shows the highest uncertainty at the medium QC level (Supplemental Table 7). At the low QC level, uncertainties were slightly elevated, reaching up to 10.7%, which is consistent with the expected increase in imprecision at low concentrations. The consistency of these findings across structurally diverse steroid hormones demonstrates the robustness of the steroid hormone panel method and supports its suitability for reliable quantification in both clinical and research applications.

The method enables reliable measurements of steroid hormones in men, women, and children with only 2% of measurements falling below the limit of detection (Table 8). A major advantage of this method is the ability to quantify estradiol in patient subpopulations with reportedly low concentrations, including the clinically relevant levels observed in postmenopausal women. For example, serum estradiol concentrations as low as 36.71 pmol/L (10 pg/mL) are accurately quantified with this method (Table 4). Other subpopulations with clinically relevant low estradiol concentrations include, but are not limited to, patients receiving hormone-suppressive or hormone-modulating therapies such as breast/prostate cancer treatment or men with age-related hypogonadism; however, samples from these groups were not tested by this method.

**Table 8:** Serum concentration ranges of eight steroid hormones across men, women, and children. Analyte concentration ranges across all groups evaluated, including males, pre- and postmenopausal females, and children all demonstrated reliable quantitation (including estradiol at ≥ 36.71 pmol/L (10 pg/mL)) and expected group- and individual-level variability. Median, interquartile ranges (25th–75th percentiles) and full ranges (minimum–maximum) are shown. The data highlight expected hormonal differences between groups as well as inter-individual variability, supporting the assay’s utility for physiological and clinical investigations

	Male (*n* = 100)		Premenopausal females, Age < 50 (*n* = 51)		Postmenopausal females, Age ≥ 60 (*n* = 49)		Children (*n* = 68)	
Analyte	N	Median (25th–75th Pct) [Min–Max]	N	Median (25th–75th Pct) [Min–Max]	N	Median (25th–75th Pct) [Min–Max]	N	Median (25th–75th Pct) [Min–Max]
17-OHP (nmol/L)	100	2.15	51	1.16	47	0.43	68	0.50
		(1.35–2.91)		(0.75–2.16)		(0.26–0.66)		(0.34–0.75)
		[0.18–5.35]		[0.21–5.56]		[0.16–10.62]		[0.10–1.94]
AD (nmol/L)	100	1.07	51	1.42	48	0.77	68	1.78
		(0.77–1.30)		(0.90–2.20)		(0.58–1.03)		(1.12–2.63)
		[0.37–2.8]		[0.21–4.58]		[0.09–15.82]		[0.27–4.74]
P4 (nmol/L)	59	0.09	46	0.30	32	0.08	68	0.10
		(0.06–0.15)		(0.13–6.34)		(0.06–0.14)		(0.08–0.13)
		[0.04–0.85]		[0.05–38.79]		[0.04–0.33]		[0.05–2.84]
TT (nmol/L)	100	14.35	51	1.05	49	0.58	68	0.40
		(10.95–18.71)		(0.71–1.33)		(0.38–0.82)		(0.28–0.58)
		[3.99–69.29]		[0.23–2.26]		[0.03–6.84]		[0.09–1.25]
E1 (pmol/L)	100	109.75	51	188.59	48	62.84	65	60.54
		(87.3–133.78)		(133.07–283.86)		(42.02–96.05)		(33.44–87.28)
		[41.72–316.33]		[36.54–611.75]		[13.89–325.54]		[14.16–160.31]
E2 (pmol/L)	98	101.92	49	291.10	38	32.36	45	72.66
		(71.95–127.66)		(157.45–483.42)		(18.18–730)		(35.64–99.62)
		[19.72–523.44]		[20.09–1068.39]		[9.95–309.67]		[18.18–229.42]
E1S (pmol/L)	100	1396.17	51	2351.24	48	512.78	68	713.34
		(947.38–2219.55)		(1201.27–4224.14)		(317.42–758.47)		(377.53–1248.12)
		[237.63–5943.49]		[236.68–12,811.36]		[43.85–1787.34]		[36.97–4431.87]
DHEAS (µmol/L)	99	4.00	51	3.58	49	1.23	68	1.79
		(2.19–5.89)		(2.26–5.67)		(0.87–1.72)		(1.09–2.30)
		[0.27–12.90]		[1.09–10.16]		[0.30–3.25]		[0.39–5.46]

**Fig. 3:**
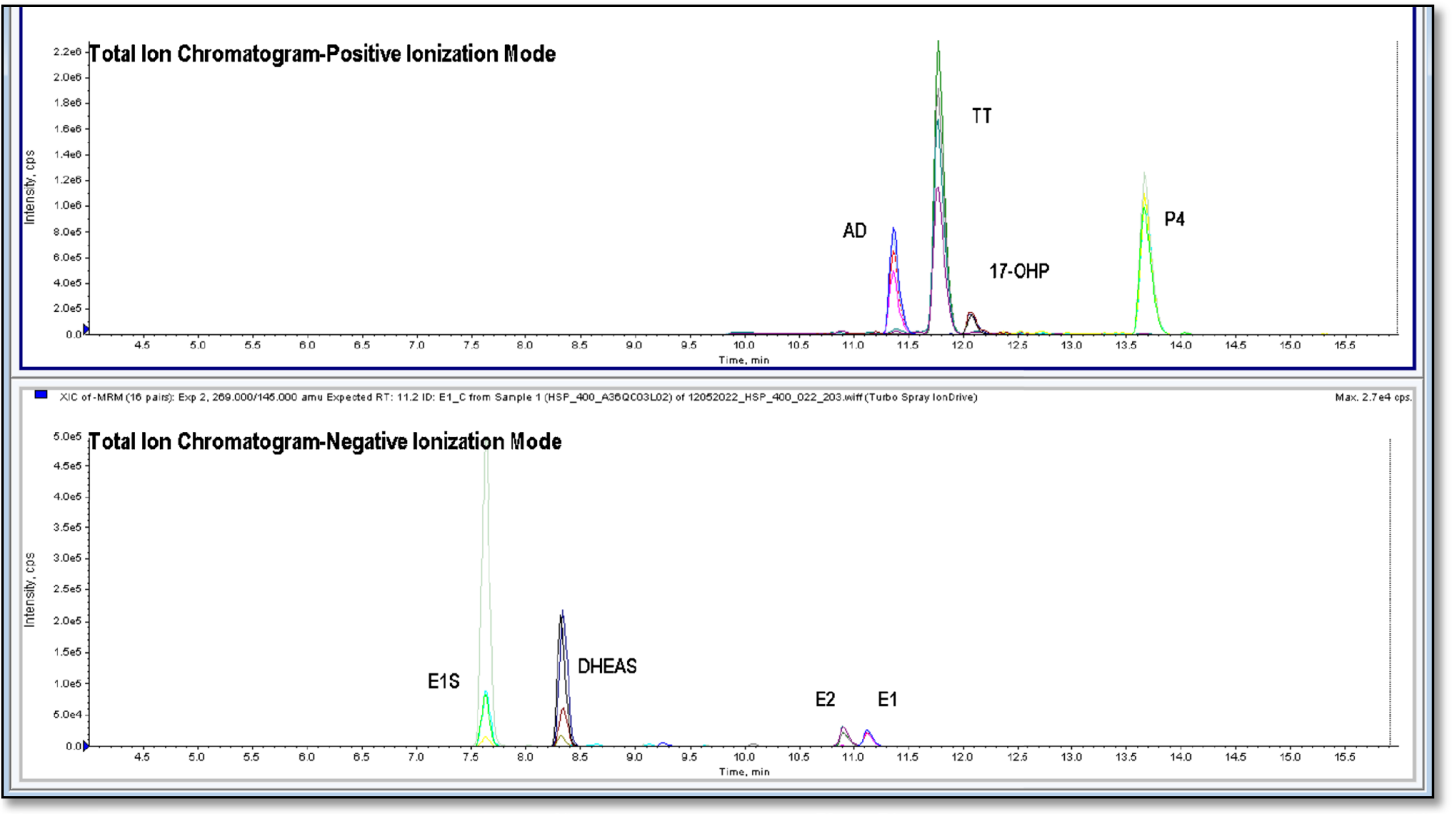
Representative LC-MS/MS chromatograms of a male sample. An example of chromatographic separation using optimized mass spectrometry parameters, which enable the simultaneous and precise quantification of eight steroid hormones. This approach facilitates accurate steroid hormone profiling in individuals and across populations. The concentration of TT in this sample is 6.9 nmol/L (198.6 ng/dL)

**Fig. 4:**
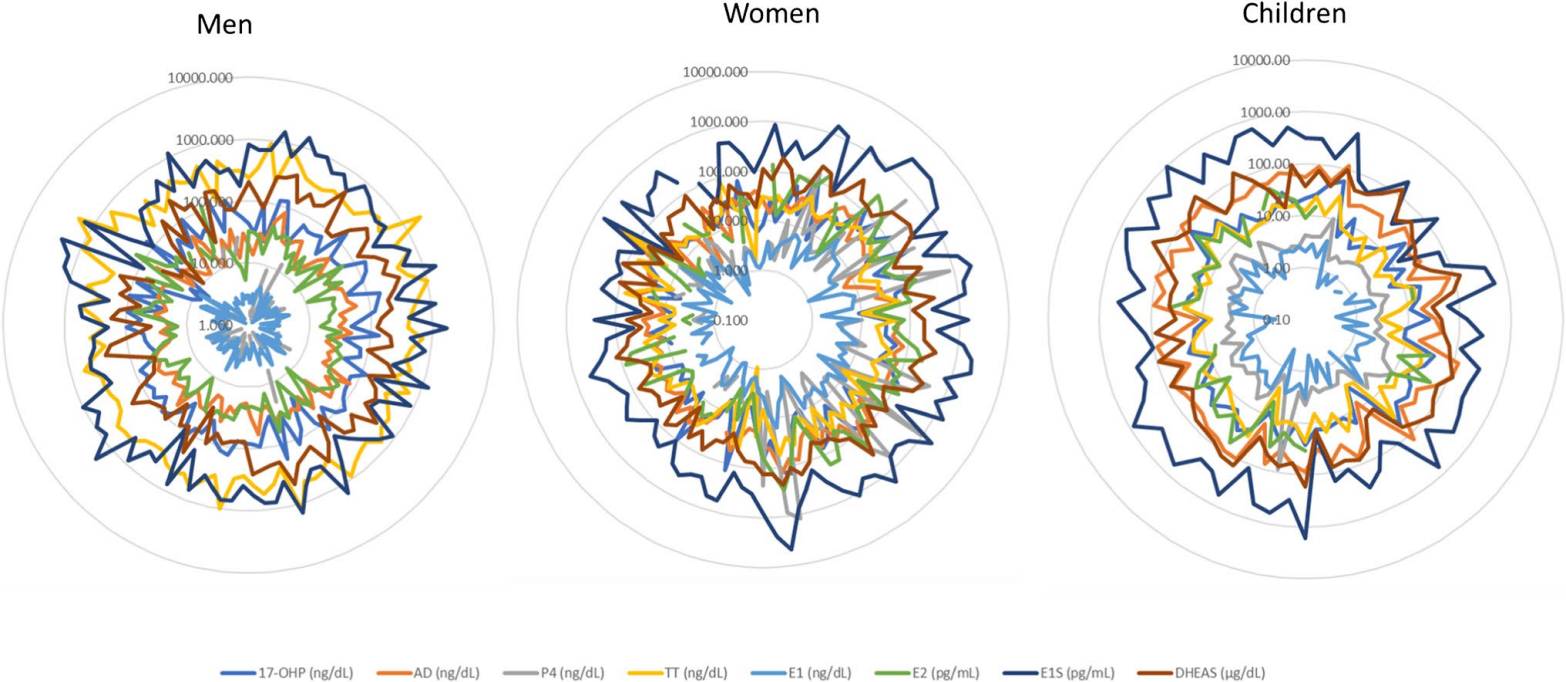
Steroid hormone concentration profiles across demographic groups. Radar plots show distributions of eight steroid hormones in men, pre- and postmenopausal women, and children. Hormone patterns differed between groups, with estradiol particularly well resolved at low concentrations, including the clinically relevant threshold of 36.7 pmol/L (10 pg/mL) in postmenopausal women. Notable inter-individual variability is observed within each group, underscoring the assay’s ability to capture both group-level differences and individual hormone profiles

Typical examples of LC-MS/MS chromatograms for male and female samples are shown in Figures 3 and Supplemental Figure 2. Appropriate chromatographic separation and optimized mass spectrometry parameters ensure simultaneous and precise quantification of each analyte, making steroid hormone profiling of individuals and the general population possible (Figure 4). As expected, hormone patterns differ across demographic groups (Figure 4), and notable inter-individual variability is observed within each group, which is subject to ongoing research. This method is a valuable tool, and its use allows one to characterize such patterns and to investigate their relationships with physiological states and environmental influences.

The clinical and research significance of this assay is substantial, particularly in oncology. Steroid hormones are increasingly recognized as potential biomarkers for certain cancers, bone diseases, and cardiovascular diseases, as well as developmental disorders [8]. The ability to measure multiple hormones simultaneously allows for a comprehensive assessment of the endocrine landscape in a patient, offering deeper insights into hormone interactions and their collective roles in disease initiation and progression. In cancer research, this approach supports mechanistic studies of carcinogenesis, identification of novel therapeutic targets, and improved biomarker discovery.

Beyond research, the method offers practical advantages for clinical use. Its efficiency and cost-effectiveness increase accessibility, while the minimally invasive nature of serum-based testing—requiring only small sample volumes—improves patient comfort and compliance. The capacity to measure eight steroid hormones simultaneously in a small volume of serum represents a significant advancement in both diagnostics and research, with the potential to expand our broader understanding of disease pathophysiology and to improve patient outcomes.

## Conclusions

The development and validation of this novel isotope-dilution UHPLC-MS/MS method for measuring eight clinically relevant steroid hormones in serum represents a significant advancement in endocrinology and metabolic disease research. Unlike conventional assays that target single analytes or require derivatization, this approach enables the simultaneous, highly accurate and precise quantification of multiple steroids from minimal serum volumes. By integrating certified reference materials, stable isotope-labeled internal standards for each analyte, and optimized chromatographic conditions, the method ensures exceptional selectivity, reproducibility, and robustness while effectively minimizing matrix effects. A particularly distinctive feature is its ability to reliably quantify low concentrations of estradiol, a challenge unmet by many existing assays and one of great clinical importance for postmenopausal women and pediatric populations. Combined with wide reportable ranges, low measurement uncertainties, and comprehensive interference testing, the method provides a uniquely powerful platform for endocrine profiling across diverse populations. Overall, this innovative assay provides a robust analytical approach that supports biomarker discovery and mechanistic research and will facilitate a more comprehensive evaluation of steroid hormone profiles in clinical and research settings. Its improved sensitivity and selectivity, combined with a broad reportable range, make it well-suited for studies investigating hormonal interactions and their roles in disease.

## Supplementary Information

Below is the link to the electronic supplementary material.

ESM 1 (DOCX 139 KB)

## Data Availability

Data are available upon request.

## Declarations

### Animal welfare

N/A

### Disclaimer

The findings and conclusions in this report are those of the author(s) and do not necessarily represent the official position of the Centers for Disease Control and Prevention/the Agency for Toxic Substances and Disease Registry. Use of trade names is for identification only and does not imply endorsement by the Centers for Disease Control and Prevention, the Public Health Service, and the US Department of Health and Human Services. This research was supported, in part, by the Intramural Research Program of the National Institutes of Health (NIH) (Z01-ES103315 and ZIC ES103363 to NDS). NDS is also supported as a Lasker Clinical Research Scholar (1SI2ES025429-01). The contributions of the NIH author(s) were made as part of their official duties as NIH federal employees, are in compliance with agency policy requirements, and are considered Works of the United States Government. However, the findings and conclusions presented in this paper are those of the author(s) and do not necessarily reflect the views of the NIH or the U.S. Department of Health and Human Services.
