# Supplementary material for: Highly specific ID-UHPLC-MS/MS method for analyzing polar and non-polar steroid hormones
Source: Anal Bioanal Chem. 2026 Apr 29;418(13):4153–68. doi: 10.1007/s00216-026-06504-3 (PMC13264578; doi:10.1007/s00216-026-06504-3)
Supplement: Supplementary file 1 — (DOCX 139 KB) [file 216_2026_6504_MOESM1_ESM.docx]

**Online Supplement**

**Highly Specific ID-UHPLC-MS/MS Method for Analyzing Polar and Non-Polar Steroid Hormones**

Lumi Duke^1^, Paul H. Kim^2^, Alicia N. Lyle^1^, Nathalie Shaw ^3^, Julianne C. Botelho ^1^, Hubert W. Vesper^1*^

^1^ Centers for Disease Control and Prevention, National Center for Environmental Health, Atlanta, GA 30341, USA;

^2^ Battelle Memorial Institute, Atlanta, GA 30345, USA;

^3^ National Institute of Health, National Institute of Environmental Health Sciences, Research Triangle Park, NC 27709, USA

**Online Supplement Contents:**

**Supplemental Table 1.** List of reference materials and their corresponding stable isotope compounds, including purity levels and sources.

**Supplemental Table 2.** List of 27 steroidal compounds utilized for interference testing.

**Supplemental Table 3A.** Calibration standards and their concentrations for each analyte in the steroid hormone method.

**Supplemental Table 3B.** Calibrator working solutions and their concentrations in SI Units (nmol/L).

**Supplemental Table 4.** Internal standard (IS) concentrations for each analyte in the steroid hormone panel method.

**Supplemental Table 5.** Mass spectrometer settings for each analyte and internal standard (IS).

**Supplemental Table 6.** Suggested analytical performance criteria for steroid hormones and desired standard relative measurement uncertainty based on biological variability.

**Supplemental Table 7.** Mean concentrations and associated standard measurement uncertainties for steroid hormones analyzed in quality control (QC) samples.

**Supplemental Figure 1.** Example of chromatographic separation of potential interfering compounds.

**Supplemental Figure 2.** Representative LC–MS/MS chromatograms of a female sample.

**Supplemental Table 1.** **List of reference materials and their corresponding stable isotope compounds, including purity levels and sources.** “-IS” denotes the corresponding stable isotope-labeled internal standard. Purity (%) represents manufacturer-reported chemical purity. Manufacturer indicates supplier and location.

| **Abbreviation** | **Calibrator and Internal Standard Material** | **Purity (%)** | **Manufacturer** |
| --- | --- | --- | --- |
| 17-OHP | 17α-Hydroxyprogesterone | 98.25 | Cerilliant (Round Rock, TX) |
| 17-OHP-IS | 17α-Hydroxyprogesterone-2,3,4-^13^C_3_ | 99.41 | Cerilliant (Round Rock, TX) |
| AD | Androstene-3,17-dione | 99.44 | Cerilliant (Round Rock, TX) |
| AD-IS | Androstene-3,17-dione-2,3,4-^13^C_3_ | 97.87 | Cerilliant (Round Rock, TX) |
| P4 | Progesterone | 99.3 | National Metrology Institute of Japan (NMIJ) |
| P4-IS | Progesterone-2,3,4-^13^C_3_ | 98.6 | IsoSciences (King of Prussia, PA) |
| TT | Testosterone | 99.2 | Australian Government National Measurement Institute (ANMI) |
| TT-IS | Testosterone-2,3,4-^13^C_3_ | 99.65 | Cerilliant (Round Rock, TX) |
| E1 | Estrone | 99.27 | Cerilliant (Round Rock, TX) |
| E1-IS | Estrone-2,3,4-^13^C_3_ | 98.10 | Cerilliant (Round Rock, TX) |
| E2 | 17β-Estradiol | 98.4 | National Metrology Institute of Japan (NMIJ) |
| E2-IS | 17β-Estradiol-2,3,4-^13^C_3_ | 92.54 | Cerilliant (Round Rock, TX) |
| E1S | Estrone 3-sulfate sodium salt | 99.0 | Cambridge Isotope Laboratories, Inc. (Andover, MA) |
| E1S-IS | Estrone 3-sulfate-13,14,15,16,17,18-^13^C_6_ sodium salt | 84.5 | Cambridge Isotope Laboratories, Inc. (Andover, MA) |
| DHEAS | Dehydroepiandrosterone 3-sulfate sodium salt | 94.36 | Cerilliant (Round Rock, TX) |
| DHEAS-IS | Dehydroepiandrosterone-D_5_-3-sulfate sodium salt | 99.34 | Cerilliant (Round Rock, TX) |

**Supplemental Table 2**. **List of 27 steroidal compounds utilized for interference testing.** Steroidal compounds frequently found in circulation were commercially sourced, as indicated, and were used to test the steroid hormone panel method for potential interferences. Compounds were tested at indicated concentrations and included isomers and compounds structurally analogous to the target hormones.

| **Compound** | **Concentration**  **(SI Units)** | **Source** |
| --- | --- | --- |
| Cortisol | 53520 nmol/L | Cerilliant (Round Rock, TX) |
| 11-Deoxycorticosterone | 24.24 nmol/L | Cerilliant (Round Rock, TX) |
| Methandienone | 19.97 nmol/L | Cerilliant (Round Rock, TX) |
| Aldosterone | 16.62 nmol/L | Cerilliant (Round Rock, TX) |
| Corticosterone | 5.78 µmol/L | Cerilliant (Round Rock, TX) |
| 11-Deoxycortisol | 26.01 nmol/L | Cerilliant (Round Rock, TX) |
| Epitestosterone | 17.35 nmol/L | Cerilliant (Round Rock, TX) |
| 17α-Methyltestosterone | 16.53 nmol/L | Cerilliant (Round Rock, TX) |
| 18-Hydroxycorticosterone | 24.84 nmol/L | Cerilliant (Round Rock, TX) |
| Nandrolone | 18.22 nmol/L | Cerilliant (Round Rock, TX) |
| Stanozolol | 15.22 nmol/L | Cerilliant (Round Rock, TX) |
| 17α-Ethynylestradiol | 3.37 nmol/L | Cerilliant (Round Rock, TX) |
| Equilin | 3.73 nmol/L | Cerilliant (Round Rock, TX) |
| 6β-Hydroxytestosterone | 16.42 nmol/L | Cerilliant (Round Rock, TX) |
| 16α-Hydroxyestrone | 17.46 nmol/L | Cerilliant (Round Rock, TX) |
| 17α-Hydroxypregnenolone | 15.05 nmol/L | Cerilliant (Round Rock, TX) |
| Prednisolone | 13.87 nmol/L | Cerilliant (Round Rock, TX) |
| Cortisone | 0.831 µmol/L | Cerilliant (Round Rock, TX) |
| 2-Hydroxyestrone | 17.46 nmol/L | Cerilliant (Round Rock, TX) |
| Trenbolone | 33.29 nmol/L | Cerilliant (Round Rock, TX) |
| Dehydroepiandrosterone | 27.76 nmol/L | Cerilliant (Round Rock, TX) |
| Dihydrotestosterone | 34.4 nmol/L | Cerilliant (Round Rock, TX) |
| Etiocholanolone | 68.86 nmol/L | National Measurement Institute, Australia (NMI) |
| Pregnenolone | 15.8 nmol/L | Sigma-Aldrich (St. Louis, MO) |
| Estriol | 10410 nmol/L | Cerilliant (Round Rock, TX) |
| Testosterone glucuronide | 538.18 nmol/L | National Measurement Institute, Australia (NMI) |
| Testosterone sulfate | 54.27 nmol/L | National Measurement Institute, Australia (NMI) |

| **Calibrator Working Solution Code** | **Concentration (*nmol/L*)** | | | | | | | |
| --- | --- | --- | --- | --- | --- | --- | --- | --- |
|  | ***17-OHP*** | ***AD*** | ***P4*** | ***TT*** | ***E1*** | ***E2*** | ***E1S*** | ***DHEAS*** |
| CC01 | 0.0238 | 0.0333 | 0.156 | 0.0806 | N/A | 0.00964 | 0.0113 | N/A |
| CC02 | 0.0892 | 0.125 | 0.584 | 0.302 | 0.0138 | 0.0361 | 0.0424 | 76.9 |
| CC03 | 0.297 | 0.416 | 1.95 | 1.01 | 0.0459 | 0.120 | 0.141 | 256 |
| CC04 | 0.595 | 0.833 | 3.89 | 2.01 | 0.0918 | 0.241 | 0.283 | 512 |
| CC05 | 1.49 | 2.08 | 9.73 | 5.04 | 0.230 | 0.602 | 0.706 | 1280 |
| CC06 | 4.46 | 6.25 | 29.2 | 15.1 | 0.689 | 1.81 | 2.12 | 3840 |
| CC07 | 5.95 | 8.33 | 38.9 | 20.1 | 0.918 | 2.41 | 2.83 | 5120 |
| CC08 | 7.73 | 10.8 | 50.6 | 26.2 | 1.19 | 3.13 | 3.67 | 6660 |
| CC09 | 9.51 | 13.3 | 62.3 | 32.2 | 1.47 | 3.85 | 4.52 | 8200 |
| CC10 | 11.9 | 16.7 | N/A | 40.3 | 1.84 | 4.82 | 5.65 | 10200 |

**Supplemental Table 3A. Calibration standards and their concentrations for each analyte in the steroid hormone method.** Ten calibration standards were generated by diluting individual stock solutions in a 20/80 (v/v) ethanol (EtOH)/water mixture to achieve the target concentrations indicated. The concentrations of the steroid hormones in the calibrator working solutions are expressed in SI Units (nM).

**Supplemental Table 3B. Calibrator working solutions and their concentrations in conventional units.**

| **Calibrator Working Solution Code** | **Concentration** | | | | | | | |
| --- | --- | --- | --- | --- | --- | --- | --- | --- |
|  | ***17-OHP (ng/dL)*** | ***AD***  ***(ng/dL)*** | ***P4***  ***(ng/dL)*** | ***TT***  ***(ng/dL)*** | ***E1***  ***(ng/dL)*** | ***E2***  ***(pg/mL)*** | ***E1S***  ***(pg/mL)*** | ***DHEAS (µg/dL)*** |
| CC01 | 0.786 | 0.954 | 4.90 | 2.32 | N/A | 2.63 | 3.96 | N/A |
| CC02 | 2.95 | 3.58 | 18.4 | 8.72 | 0.372 | 9.84 | 14.9 | 2.83 |
| CC03 | 9.83 | 11.9 | 61.2 | 29.1 | 1.24 | 32.8 | 49.5 | 9.44 |
| CC04 | 19.7 | 23.9 | 122 | 58.1 | 2.48 | 65.6 | 99.0 | 18.9 |
| CC05 | 49.1 | 59.6 | 306 | 145 | 6.21 | 164 | 248 | 47.2 |
| CC06 | 147 | 179 | 918 | 436 | 18.6 | 492 | 743 | 142 |
| CC07 | 197 | 239 | 1220 | 581 | 24.8 | 656 | 990 | 189 |
| CC08 | 255 | 310 | 1590 | 755 | 32.3 | 853 | 1290 | 245 |
| CC09 | 314 | 382 | 1960 | 930 | 39.7 | 1050 | 1580 | 302 |
| CC10 | 393 | 477 | N/A | 1160 | 49.7 | 1310 | 1980 | 378 |

**Supplemental Table 4. Internal standard (IS)** **concentrations for each analyte in the steroid hormone panel method.**  IS working solutions were prepared by diluting individual isotopically labeled standards in ethanol (EtOH) and combining them into a single solution. Concentrations are reported in both SI units and conventional units.

| **Analyte** | **Concentration** |
| --- | --- |
| 17-OHP nmol/L (ng/dL) | 2.27 (75) |
| AD nmol/L (ng/dL) | 3.49 (100) |
| P4 nmol/L (ng/mL) | 15.9 (5) |
| TT nmol/L (ng/dL) | 13.9 (400) |
| E1 pmol/L (pg/mL) | 1849 (500) |
| E2 pmol/L (pg/mL) | 4589 (1250) |
| E1S pmol/L (pg/mL) | 10920 (4000) |
| DHEAS µmol/L (µg/dL) | 0.102 (3.75) |

**Supplemental Table 5. Mass spectrometer settings for each analyte and internal standard (IS).** Mass spectrometer settings for each analyte in the steroid hormone panel method are provided, including electrospray ionization mode (ESI Mode), collision energy (CE), declustering potential (DP), and quantifier and qualifier m/z values optimized for each compound.

| **Analyte** | **ESI Mode** | **Analyte CE**  **(QI / CI) (V)** | **Analyte DP (V)** | **Analyte**  ***m/z (QI, CI)*** | **IS**  ***m/z (QI, CI)*** |
| --- | --- | --- | --- | --- | --- |
| 17-OHP | + | 31 / 33 | 11 | 331.2 > 97, 109 | 334.2 > 100, 112 |
| AD | + | 27 / 29 | 31 | 287.2 > 97, 109 | 290.3 > 100, 112 |
| P4 | + | 29 / 31 | 16 | 315.2 > 97, 109 | 318.2 > 100, 112 |
| TT | + | 25 / 25 | 190 | 289.3 > 97, 109 | 292.3 > 100, 112 |
| E1 | - | -51 / -51 | -90 | 269.1 > 145, 143 | 272.0 > 148, 146 |
| E2 | - | -50 / -52 | -40 | 271.1 > 145, 183 | 274.1 > 148, 186 |
| E1S | - | -44 / -66 | -55 | 349.0 > 269, 145 | 353.1 > 273, 147 |
| DHEAS | - | -60 / -20 | -45 | 367.1 > 80, 97 | 372.1 > 80, 98 |

**Supplemental Table 6. Suggested analytical performance criteria for steroid hormones and desired standard relative measurement uncertainty based on biological variability.**

| **Analyte** | **Accuracy (%)** | **Imprecision (%)** | **Total Error (%)** | **Minimum Standard Measurement Uncertainty (%)** | **Desirable Standard Measurement Uncertainty (%)** | **Source^d^** |
| --- | --- | --- | --- | --- | --- | --- |
| 17-OHP | 11.9 | 14.2 | 35.3 | 21.2 | 14.2 | [EFLM](https://www.westgard.com/biodatabase1.htm) Biological Variation Database ^a^ |
| AD | 10.5 | 7.9 | 23.5 | 11.85 | 7.9 | [Westguard](https://www.westgard.com/biodatabase1.htm) Biological Variation Database ^b^ |
| P4 | 15 | 15 | 39.8 | 15 | 15 | FDA Bioanalytical Method Validation Guidance for Industry ^c^ |
| TT | 6.2 / 6.4 | 6.3 / 4.63 | 16.5 / 14.0 | 9.4/ 4.63 | 6.3/ 4.63 | [EFLM](https://www.westgard.com/biodatabase1.htm) Biological Variation Database / PATH |
| E1 | 15 | 15 | 39.8 | 15 | 15 | FDA Bioanalytical Method Validation Guidance for Industry |
| E2 | 5.0 / 12.5 | 7.5 | 17.3 / 24.9 | 11.3 | 7.5 | [EFLM](https://www.westgard.com/biodatabase1.htm) Biological Variation Database / HoSt |
| E1S | 15 | 15 | 39.8 | 15 | 15 | FDA Bioanalytical Method Validation Guidance for Industry |
| DHEAS | 5.5 | 3.0 | 10.3 | 4.5 | 3.0 | [EFLM](https://www.westgard.com/biodatabase1.htm) Biological Variation Database |

^a^ <https://biologicalvariation.eu/> (accessed on: 02/15/2023)

^b^ <https://www.westgard.com/biodatabase1.htm> (accessed on: 02/15/2023)

^c^ Bioanalytical Method Validation Guidance for Industry, D.o.D.I. Office of Communications, Center for Drug Evaluation and Research, Editor. 2018, Food and Drug Administration.

^d^ When performance criteria are derived from biological variability, desirable criteria are listed.

**Supplemental Table 7.** **Mean concentrations and associated standard measurement uncertainties for steroid hormones. S**teroid hormone concentrations were determined for quality control (QC) samples at high, medium, and low concentrations. Concentrations are expressed in SI Units, with approximate conventional mass unit conversions provided in parentheses. Standard measurement uncertainty is presented as a percentage.

| **Sample Description** | **Mean Concentration (n = 204)** | | | | | | | |
| --- | --- | --- | --- | --- | --- | --- | --- | --- |
|  | 17-OHP  nmol/L (ng/dL) | AD  nmol/L (ng/dL) | P4  nmol/L (ng/mL) | TT  nmol/L (ng/dL) | E1  pmol/L (pg/mL) | E2  pmol/L (pg/mL) | E1S  pmol/L (pg/mL) | DHEAS  µmol/L (µg/dL) |
| QC High | 3.69 (122) | 9.70 (278) | 23.8 (7.50) | 20.9 (603) | 1715 (464) | 3111 (847) | 1696 (621) | 3.22 (119) |
| QC Medium | 1.25 (41.3) | 3.01 (86.1) | 7.67 (2.41) | 7.07 (204) | 621 (168) | 990 (270) | 568 (208) | 1.20 (44.4) |
| QC Low | 0.26 (8.64) | 0.56 (16.1) | 1.47 (0.46) | 1.35 (38.9) | 119 (32.3) | 192 (52.4) | 105 (38.4) | 0.23 (8.40) |
|  |  |  |  |  |  |  |  |  |
|  | **Standard Measurement Uncertainty, %^a^** | | | | | | | |
|  | 17-OHP | AD | P4 | TT | E1 | E2 | E1S | DHEAS |
| QC High | 5.1 | 4.1 | 4.8 | 3.7 | 4.2 | 5.5 | 9.3 | 7.0 |
| QC Medium | 5.4 | 3.5 | 5.1 | 3.7 | 5.9 | 5.3 | 9.8 | 6.9 |
| QC Low | 9.0 | 4.6 | 6.5 | 4.8 | 7.7 | 6.5 | 8.3 | 10.7 |

^a^ Expanded measurement uncertainty (U) at 95.45% level of confidence can be calculated by multiplying by a coverage factor of 2.

**Supplemental Figure 1.** **Example of chromatographic separation of potential interfering compounds.** The chromatogram shows that two possible interfering compounds: epitestosterone (positive mode) and aldosterone (negative mode) are resolved from the steroids measured by this panel method. Distinct retention times and the absence of peak overlap confirm that these compounds do not interfere with analyte separation.


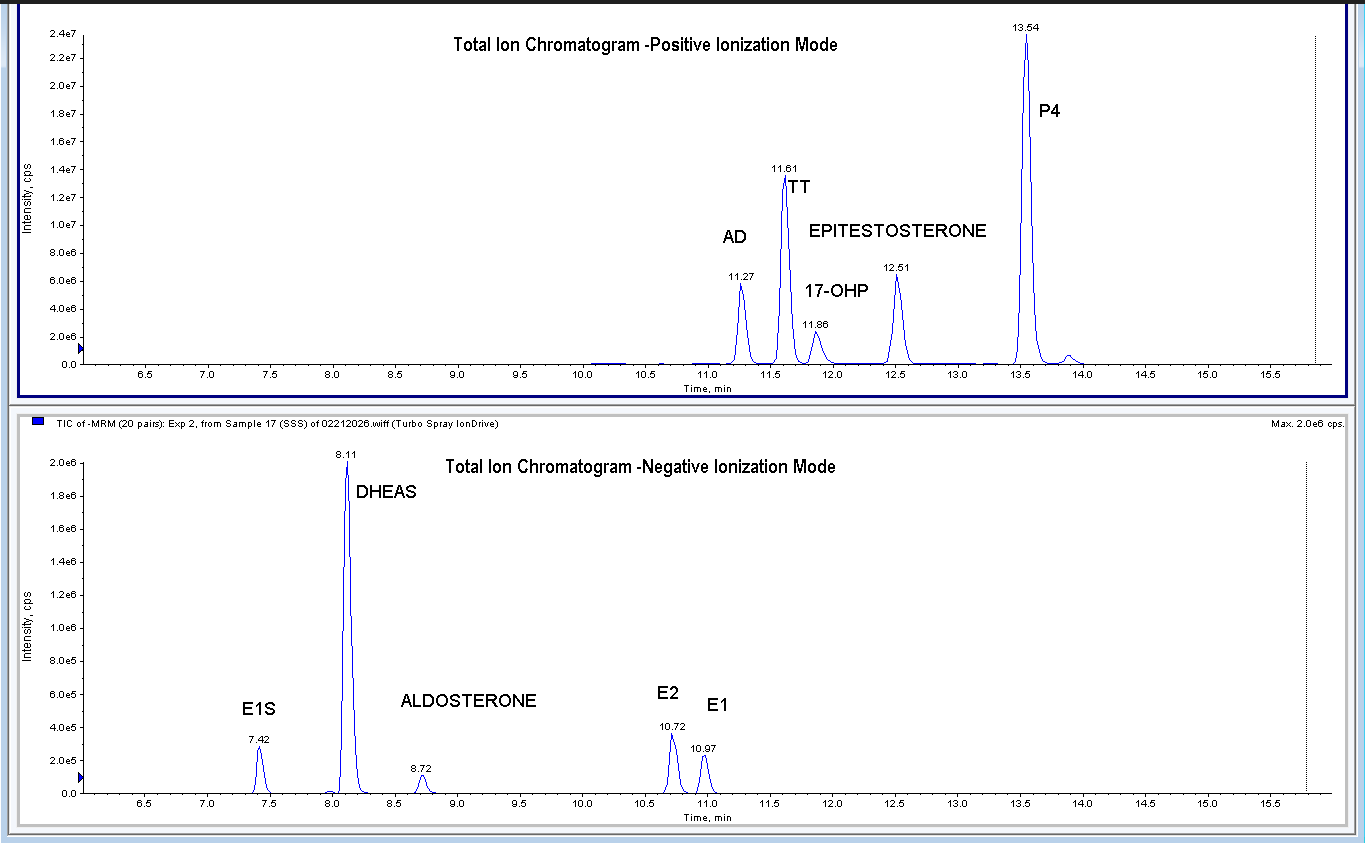


**Supplemental Figure 2. Representative LC–MS/MS chromatograms of a female sample.** Example of chromatographic separation using optimized mass spectrometry parameters, which enables the simultaneous and precise quantification of eight steroid hormones. This approach facilitates accurate steroid hormone profiling in individuals and across populations. The concentration of TT in this sample is 0.57 nmol/L (16.5 ng/dL).


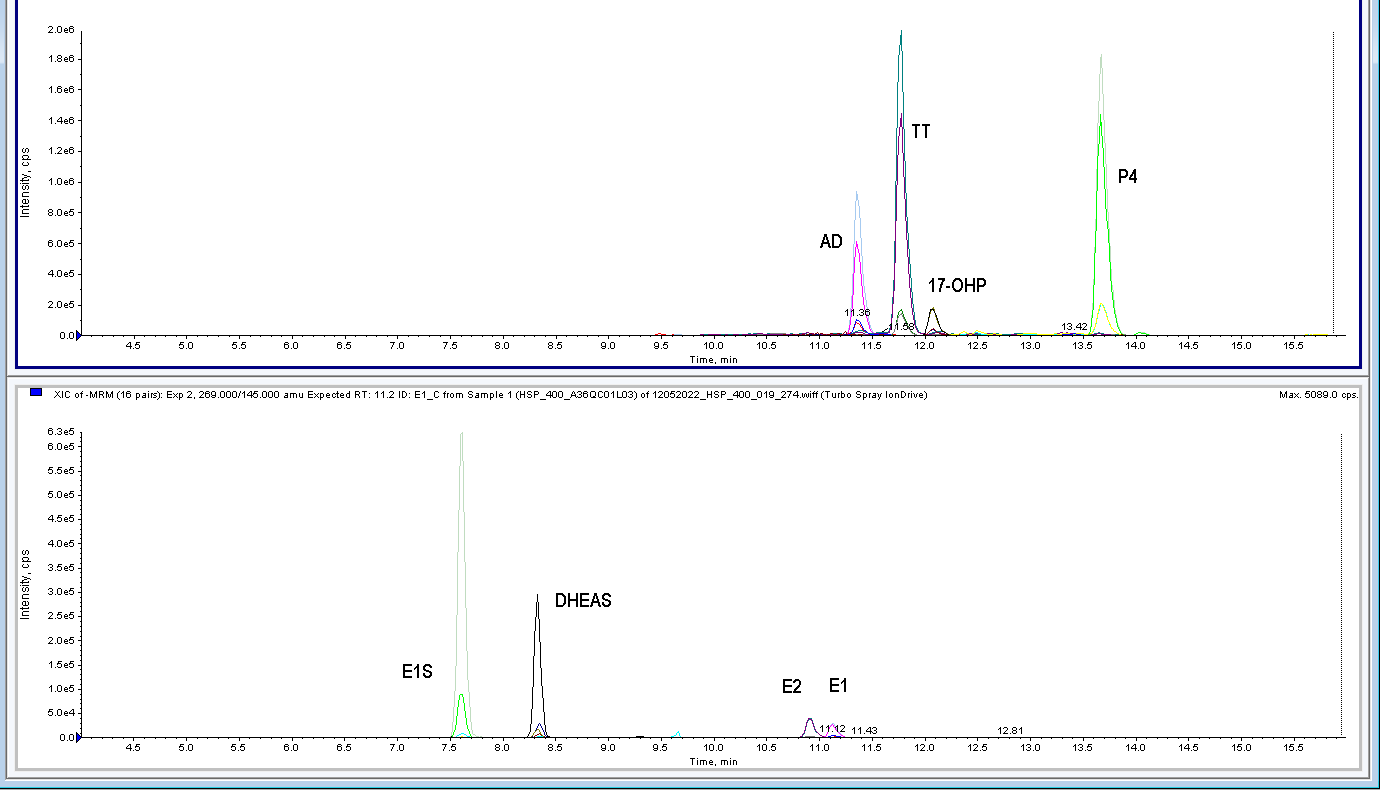


**Total Ion Chromatogram-Positive Ionization**

**Total Ion Chromatogram-Positive Ionization**
